# Mind Perception in HRI: Exploring Users’ Attribution of Mental and Emotional States to Robots with Different Behavioural Styles

**DOI:** 10.1007/s12369-023-00989-z

**Published:** 2023-03-26

**Authors:** Ilenia Cucciniello, Sara Sangiovanni, Gianpaolo Maggi, Silvia Rossi

**Affiliations:** 1 Department of Electrical Engineering and Information Technologies, University of Naples Federico II, Via Claudio 80, 80125 Naples, Italy; 2 Department of Psychology, University of Campania Luigi Vanvitelli, Viale Ellittico, 31, 81100 Caserta, Italy

**Keywords:** Mind attribution, Interaction style, Robot behaviour

## Abstract

Theory of Mind is crucial to understand and predict others’ behaviour, underpinning the ability to engage in complex social interactions. Many studies have evaluated a robot’s ability to attribute thoughts, beliefs, and emotions to humans during social interactions, but few studies have investigated human attribution to robots with such capabilities. This study contributes to this direction by evaluating how the cognitive and emotional capabilities attributed to the robot by humans may be influenced by some behavioural characteristics of robots during the interaction. For this reason, we used the Dimensions of Mind Perception questionnaire to measure participants’ perceptions of different robot behaviour styles, namely Friendly, Neutral, and Authoritarian, which we designed and validated in our previous works. The results obtained confirmed our hypotheses because people judged the robot’s mental capabilities differently depending on the interaction style. Particularly, the Friendly is considered more capable of experiencing positive emotions such as Pleasure, Desire, Consciousness, and Joy; conversely, the Authoritarian is considered more capable of experiencing negative emotions such as Fear, Pain, and Rage than the Friendly. Moreover, they confirmed that interaction styles differently impacted the perception of the participants on the Agency dimension, Communication, and Thought.

## Introduction

Social and service robots are autonomous agents that operate in human populated environments. They must plan their activities to coordinate safely and efficiently with their human teammates [7, 32]. The ability to create correct mental models of other interacting partners provides a mechanism for achieving fluent and effective teamwork [42]. Moreover, according to [45], correctly perceiving others’ minds is a key requirement for understanding, predicting, and controlling the behaviour of interacting partners, and for establishing a social bond with them.

The ability to understand and predict others’ behaviour represents the cornerstone of most social interactions [19]. Once we have a correct mental model of interacting partners, our interactions with them can become more effective and socially accepted [45]. Since it is not possible to have direct information about others’ mental states, mind attribution requires a leap from observable behaviour to unobservable mental states [10]. Mind attribution is in the “eye of the beholder”. Therefore, in Human–Robot Interaction (HRI), some authors have started to investigate the features of the robot that may trigger the perception of intentionality and goal directed behaviors. It was found that different robot design mechanisms could be used to trigger it [5]. The human likeness of a robot, for example, may also contribute to mind attribution by simulating facial features or movements [14, 16].

However, the behavioural characteristics that may elicit mind attribution to the robot by the interacting human have not yet been sufficiently investigated. According to [28], only a few studies investigated the impact of individual differences on the attribution of anthropomorphism in robots, whereas also individual dispositional factors of the subjects may have an impact on the result. Moreover, beyond design and anthropomorphic appearance, different behavioural features may also play an important role in mind attribution toward a robot [35]. For example, being unpredictable [45] or the lack of performance [26] is associated with more perceived intentionality.

This study contributes to this direction by evaluating the attribution of cognitive and emotional capabilities to the robot as influenced by different interacting styles or behaviours it shows during the interaction. In HRI literature, it emerges that the design of the robot’s personality or, more generally, interaction style with the users may affect how people judge its behaviour [21], the development of uncanny feelings [30], and the perception of the social intelligence of the robot [23]. Hence, a robot’s interaction style may also have a potential impact on mind attribution. In previous works [8, 21, 36], we designed and validated three different robot interaction styles (*Friendly*, *Neutral*, and *Authoritarian*) that we designed by modelling verbal and non-verbal behaviours of a robot (such as tone of voice, speech speed, gestures, eye colours, feedback). Hence, here we evaluated how different interaction styles of the robot impact participants’ robot mind attribution. Understanding how people judge a robot’s mental abilities based on its behaviour could represent a starting point to better understanding whether robots can use such interaction capabilities to improve the naturalness and legibility of the interaction.

We asked the participant to complete the Dimensions of Mind Perception questionnaire [12] adapted for comparing two styles of interaction at a time on 18 mental capacities related to different mind dimensions: Experience and Agency. Particularly, Experience refers to the character’s perceived ability for “feeling” (e.g., to feel pain, rage, joy); conversely, Agency refers to the character’s perceived ability for “doing” (e.g., to plan, though, communicate). This differentiation is related to the two subcomponents of the Theory of Mind (ToM) construct: the ‘cognitive’ ToM defines a cognitive understanding of the difference between the speaker’s knowledge and that of the listener (knowledge about beliefs), instead, the ‘affective’ ToM defines the empathic recognition of the observed person’s emotional state (knowledge about emotions) [40].

Moreover, we aim also to assess whether the humans’ perception of a robot’s abilities may be related to Trust and/or the robot’s characteristics such as Anthropomorphism, Animation, Likeability, Perceived Intelligence, and Perceived Safety. Hence, the participants were asked to complete the Godspeed [4] questionnaire and the Trust Perception Scale-HRI [38]. As mentioned in [27], trust is a critical issue in human–robot interactions as it is the core of the human desire to accept and use a non-human agent and depends on our representation of this agent’s actions, beliefs, and intentions. Mental models promote trust and reliability by alleviating uncertainty in roles, responsibilities, and capabilities while working in a team [42].

## Background and Related Works

In HRI literature, the concept of mental modelling is often linked with another important concept in psychology: the “Theory of Mind” (ToM) [42]. ToM has been defined as the cognitive ability to infer own and other’s mental and emotional states (i.e., beliefs, desires, thoughts, emotions) [2]. The attribution of mental states to inanimate objects, such as virtual agents, has been used to “describe” and “design” the behaviour of such complex systems whenever their design or physical description was too complex to be easily understood [9]. On the contrary, in the HRI field, several studies are starting to investigate whether human beings attribute mental states to a robot while interacting with it or simply observing its behaviour [31]. Some researchers investigated users’ perception of the mental abilities that a robot showed during the interaction and the possible links with factors such as pleasure, acceptance, etc. For example, in [12] the robot character was rated a moderate score for the agency, higher than animals (i.e., dog, frog, and chimp), but the lowest equal with God for the experience.

Indeed, mind attribution to robots has been related to the perceived physical presence [3, 15] and anthropomorphism [5] that can be modulated by simulating human facial features and movements [14, 16]. In [17], the authors asked the question ‘Why and under which circumstances do we attribute human-like properties to machines?’. To explore this aspect, they have implemented an interactive human–robot game, introducing an increase in human similarity degrees for the gaming partner. They have shown that the tendency to build a model of another’s mind linearly increases with its perceived human likeness.

Beyond physical appearance, also social context [6] and individual differences may affect the degree of anthropomorphism attribution in robots and, consequently, of mind attribution [28]. For example, recent research has suggested that the decrease in the scores of mind perception could be related to the degree of causal attribution to the partner agent in a repeated game [24]. Moreover, Stafford et al. [41] examined predictors of robot use in a retirement village over two weeks and predicted that residents who chose to use the robot had more computer knowledge, held more positive attitudes towards robots, and attributed less mind agency to robots. Conversely, in [1], which investigated the differences between elderly and young adults in ascribing mind perception to a sociable humanoid robot, they found that the elderly attributed higher scores of mind perception to the robot, whereas young adults seemed to have a more positive attitude towards it.

In this work, we are interested in investigating whether, beyond design and anthropomorphic appearance, different behavioural features may play an important role in mind attribution toward a robot [35]. For example, the perceived transparency about robots’ machine nature could influence people’s perception [43]. As confirmation, in [44], the authors identified two factors that could potentially influence mind perception and moral concern in robots: framing (how the robot is introduced) and social behaviour (how the robot acts). Indeed, the way a robot acts, interacts, and modulates its behaviour has an impact on how the robot and the interaction are perceived [21] and so, potentially, also on mind attribution [46]. For example, in [39], participants engaged in a hallway navigation task with a robot over several trials. The display of the robot and its proxemics behaviour was manipulated, and participants made mental state attributions across trials. Results show that proxemics behaviour and robotic display characteristics differently influence the degree to which individuals perceive the robot when making mental state attributions about themselves or others. Moreover, the unpredictability of behaviour corresponded to a greater attribution of mental capabilities [46].

**Fig. 1:**
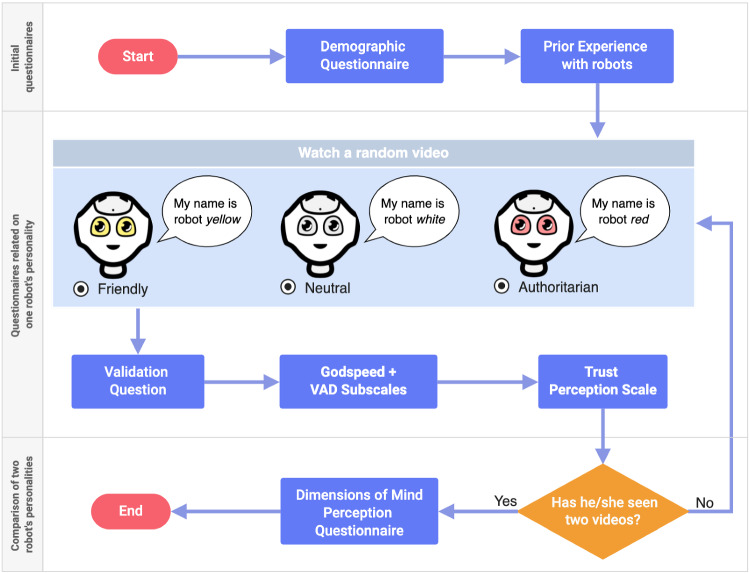
Illustration of the online survey

Gray et al. [15] conducted three studies suggesting that the higher experience perceived in a robot, rather than the agency, may be tied to feelings of users’ unease. In [25], the authors constructed two different personalities for a humanoid robot, namely a socially engaged personality that maximised its user interaction (i.e., an emotional and more interactive character) and a competitive personality that was focused on playing and winning the game (i.e., a rational character). Like our study, they assessed each personality with the Godspeed Questionnaire and the Mind Perception Questionnaire. However, no significant differences were found in the Mind Perception results. The researchers of [13] performed a study on the perception of a robot’s characteristics (i.e., anthropomorphism, animation, likeability, perceived intelligence, and perceived safety) and the changes after passive and active interaction with a physically present humanoid robot highlighting that the perception changes mainly after the first interaction.

## Materials and Methods

We created three online studies using a free web-based application. Each survey has the same structure (see Fig. 1) but compares two different robot behaviour styles: *Neutral-Authoritarian* (Survey 1), *Neutral-Friendly* (Survey 2), and *Friendly-Authoritarian* (Survey 3). This is to better highlight differences in each robot’s behaviour style compared to the other two. To reach a larger audience, we shared each survey on different social media channels, to avoid overlapping of participants, without providing monetary incentives.

**Fig. 2:**
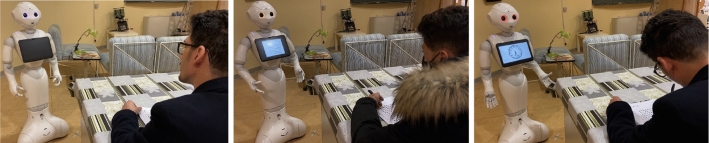
Screenshots from *Neutral* (left), *Friendly* (centre), and *Authoritarian* (right) video

### Robot’s Behaviour Styles

In our previous works [20, 21, 36], we explored the impact of different behaviours on human–robot interaction. We designed three different robot behaviour styles called *Friendly*, *Neutral*, and *Authoritarian*. Each behaviour is shaped by verbal and non-verbal interaction features like gaze, speed of speech, tone of voice, gestures, proxemics, etc. In [8], we evaluated that each simulated behaviour is correctly perceived by the participants. Hence, here we decided to use these behavioral styles to evaluate the possible different mental states attributed to the robot during the interaction. In detail, each behavioural style is characterised as follows:

- *Eye colour* For the *Friendly* robot, we used yellow colour as it causes positive emotions like joy; for the *Neutral*, we used white colour as it is not associated with emotions and for the *Authoritarian*, we used red colour as it is associated with emotions such as anger.
- *Gaze* In the *Friendly* condition, the robot’s gaze is addressed to the user during the interaction and diverted during the execution of the exercises; in the *Neutral*, the robot’s gaze is always distracted, while in the *Authoritarian*, the robot’s gaze is always directed at the user.
- *Feedback* The *Friendly* robot uses encouragements (e.g., ‘good job’, ‘do continue like this’); the *Neutral* does not use voice signals, while the *Authoritarian* uses attention recalls (e.g., ‘time is running out’, ‘are you done?’).
- *Gestures* The *Friendly* robot uses gestures to accompany the rhythm of speech; the *Neutral* robot does not use specific gestures but only a movement of simple involuntary breathing; the *Authoritarian* robot uses semantic gestures to emphasise commands.
- *Speed/Pitch* For the *Friendly*, we used a high speed in speech (85%) and a low tone of voice (50%), for the *Neutral*, we used a high speed in speech (90%) and a high tone of voice (100%), and finally for the *Authoritarian*, we used a high speed in speech (85%) and a medium-high tone of voice (75%).
- *Language* The *Friendly* robot uses informal language while the *Neutral* and the *Authoritarian* use formal language.

For a detailed explanation of the design choice please refer to [21].

### Experimental Hypotheses

We set up our experimental design and evaluation from three main hypotheses:

- *H1* The different interaction styles of the robot could have an impact on participants’ perception of the Experience dimension of its Mind Attribution. Particularly, since the *Friendly* behaviour was designed to look joyful and put the participant at ease, we hypothesised obtaining a higher number of participants that judged it as more capable of experiencing positive emotions such as Joy and Pleasure than the *Neutral* and the *Authoritarian*. Conversely, since the *Authoritarian* behaviour was designed to express authority and with a dominant appearance, we hypothesised obtaining a higher number of participants that judged it as more capable of experiencing negative emotions such as Rage and Pride than the *Neutral* and the *Friendly* (confirmatory hypothesis).
- *H2* The different interaction styles of the robot could have a different impact on participants’ perception of the Agency dimension of its Mind Perception (exploratory hypothesis).
- *H3* The robot’s mental abilities perceived by participants might be differently related to the perceived trust level in the robot and/or to the characteristics highlighted in the Godspeed questionnaire, i.e., Anthropomorphism, Animation, Likeability, Perceived Intelligence, and Perceived Safety (exploratory hypothesis).

**Table 1:** Subscales VAD added to the Godspeed questionnaire

Valence	Arousal	Dominance
Cold/Warm	Tired/Energetic	Hesitating/Determined
Hostile/Friendly	Asleep/Awake	Insignificant/Authoritative
Rude/Kind	Weak/Vigorous	Undecided/Assertive
Unfriendly/Loving	–	Submissive/Dominant

### Survey

The online survey started with a digital consent form describing the nature of the study. Individuals agreed to participate after reading and accepting the digital informed consent. We asked the participant to complete an anonymous demographic questionnaire, to collect information such as age, sex, employment status, and the prior experience questionnaire with robots [37], to find out if the participant has ever interacted with, built, or controlled a robot.

For each couple of videos, the order of presentation was randomised to balance the number of participants that watched one video first or the other (e.g., for Survey 1: *Neutral-Authoritarian* vs. *Authoritarian–Neutral*). In each video, the robot Pepper interacts by carrying out the same cognitive test with a male actor but with a different behaviour style (see Fig. 2). The robot gives the instructions of an exercise, also used in our previous studies, namely the Attentive Matrices, allowing him to complete each matrix in 15 s. Meanwhile, the robot can provide vocal feedback (such as encouragements or recalls) or not.

We designed the interaction so that the robot introduced its name after completing the cognitive task. This is to evaluate if the participant watched the video until the end. For this reason, after watching each video, we introduced a “validation question” asking the participant the robot’s name. In addition, the participant must complete:

- The Godspeed questionnaire [4]. It is used to investigate how the participant evaluated the robot on aspects like anthropomorphism, animation, perceived intelligence, likeability, and perceived safety. In addition, we defined three subscales (see Table 1) for the Valence-Arousal-Dominance (VAD) model [33] to measure perceived emotional states associated with the robot’s performance. The participant provided a score between 1 and 5 for each feature of the subscales, where the first attribute corresponds to the value 1, while the second attribute corresponds to the value 5.
- The Trust Perception subscale-HRI [37, 38]. It is made up of only 14 items and is used to investigate how much the participant trusted the robot. The participant provided a percentage score between 0% and 100% for each item of the subscale.

Finally, after watching the two videos and evaluating the individual behaviours, participants were also asked to compare the two styles of interaction through the Dimensions of Mind Perception questionnaire [12].

### Dimensions of Mind Perception Questionnaire

The Dimensions of Mind Perception questionnaire [12] was employed to assess participants’ perceptions of the robot mind. To directly compare the two behaviours on each dimension of the Mind Perception questionnaire, the original scoring system proposed by Gray et al. [12] was adapted as follows: participants were asked to choose which one of the two proposed interaction styles better reflected each dimension of the Mind Perception questionnaire. These mind perception dimensions refer to the capacity to feel Hunger, Fear, Pain, Pleasure, Rage, Desire, Personality, Consciousness, Pride, Embarrassment, and Joy, belonging to the Experience domain (the ability of “feeling”); conversely, the capacity for Self-control, Morality, Memory, Emotion recognition, Planning, Thought, and Communication belonged to the Agency domain (the ability of “doing”). For each dimension, we evaluated the percentage of time the behavioural style was selected, with respect to the other, as better reflecting such capability.

We then computed two subscores for Experience and Agency domains by summing a point whenever the interaction style was selected for that dimension. For example, in the case the Friendly, in comparison to the Neutral interaction style, obtained preferences on 3 dimensions (e.g., Desire, Personality, and Joy) out of 11 of the Experience domain, we computed an Experience score of 3 for Friendly and, consequently, a score of 8 for Neutral interaction style. The resulting values are the averages among the participants’ scores.

### Participants

We recruited a total of 175 participants aged between 18 and 67 years. We eliminated 29 of these since they did not pass the validation questions, which checked if they have correctly heard the name of the robot in the video. Hence, we considered data from 146 participants, of which 78 were males and 68 were females. Particularly, *Survey 1* was completed by 52 participants, of which 9 were eliminated, for a total of 43 participants (22 males, 21 females). *Survey 2* was completed by 66 participants, of which 6 were eliminated, for a total of 60 participants (29 males, 31 females). Finally, *Survey 3* was completed by 57 participants, of which 14 were eliminated, for a total of 43 participants (27 males, 16 females).

**Table 2:** Comparisons of dimensions of mind perception scores between authoritarian and neutral interaction styles

	Authoritarian	Neutral	$\chi ^2$/Z	*p*
*Experience*				
Hunger	27 (62.8$\%$)	16 (37.2$\%$)	2.814	0.093
Fear	18 (41.9$\%$)	25 (58.1$\%$)	1.140	0.286
Pain	16 (37.2$\%$)	27 (62.8$\%$)	2.814	0.093
Pleasure	23 (53.5$\%$)	20 (46.5$\%$)	0.209	0.647
Rage	27 (62.8$\%$)	16 (37.2$\%$)	2.814	0.093
Desire	26 (60.5$\%$)	17 (39.5$\%$)	1.884	0.170
Personality	**36 (83.7**$\%$)	7 (16.3$\%$)	19.558	<**0**.**001**
Consciousness	**33 (76.7**$\%$)	10 (23.3$\%$)	12.302	<**0**.**001**
Pride	29 (67.4$\%$)	14 (32.6$\%$)	5.233	0.022
Embarrassment	13 (30.2$\%$)	30 (69.8$\%$)	6.721	0.010
Joy	23 (53.5$\%$)	20 (46.5$\%$)	0.209	0.647
*Agency*				
Self-control	24 (55.8$\%$)	19 (44.2$\%$)	0.581	0.446
Morality	18 (41.9$\%$)	25 (58.1$\%$)	1.140	0.286
Memory	26 (60.5$\%$)	17 (39.5$\%$)	1.884	0.170
Emotion recognition	23 (53.5$\%$)	20 (46.5$\%$)	0.209	0.647
Planning	23 (53.5$\%$)	20 (46.5$\%$)	0.209	0.647
Communication	23 (53.5$\%$)	20 (46.5$\%$)	0.209	0.647
Thought	25 (58.1$\%$)	18 (41.9$\%$)	1.140	0.286
*Subscores*				
Experience subscore	6.30 ± 1.83	4.70 ± 1.83	$-$2.968	0.003
Agency subscore	3.74 ± 2.17	3.23 ± 2.17	$-$0.763	0.445

Values are expressed in n (frequency) or mean (SD). Significant values after Bonferroni’s correction are reported in bold. Values are shown as frequencies (percentages) or mean ± SD

### Statistical Analysis

We evaluated the internal consistency of the new Goodspeed-VAD by considering the Cronbach’s alpha coefficient with a value $\alpha \ge$ 0.70 [22]. The Chi-square ($\chi ^2$) goodness of fit test was applied to compare the observed frequencies on each mental capacity of the Dimensions of Mind Perception questionnaire. The two aggregated dimensions of mind (i.e., Experience and Agency) were compared using the non-parametric Wilcoxon signed-rank test. We applied Bonferroni’s correction for multiple comparisons (i.e., $\alpha / k$ where *k* is the number of comparisons) to control for the type I error; thus, *p* values <0.002 (0.05/20) were considered significant. The same statistical analyses were carried out for each survey. Moreover, we performed Spearman’s rank correlation to evaluate the associations between Experience and Agency dimensions with Trust and Godspeed variables codified for each interaction style using a composite dataset. The critical alpha level was set at.05 for correlation analyses. All analyses were performed with IBM SPSS-26.

## Results

The Goodspeed-VAD demonstrated excellent internal consistency as shown by Cronbach’s alpha of 0.950 for the Neutral interaction style, 0.969 for the Friendly interaction style, and 0.948 for the Authoritarian interaction style. In the following, we present our results on mind attribution using the dimension of mind perception questionnaire on each pair of considered interaction styles. We discuss the possible associations of different mind perception results and Goodspeed-VAD and Trust evaluation.

**Table 3:** Comparisons of dimensions of mind perception scores between friendly and neutral interaction styles

	Friendly	Neutral	$\chi ^2$/Z	*p*
*Experience*				
Hunger	**53 (88.3**$\%$)	7 (11.7$\%$)	35.267	<**0**.**001**
Fear	14 (23.3$\%$)	**46 (76.7**$\%$)	17.067	<**0**.**001**
Pain	20 (33.3$\%$)	40 (66.7$\%$)	6.667	0.010
Pleasure	**56 (93.3**$\%$)	4 (6.7$\%$)	45.067	<**0**.**001**
Rage	13 (21.7$\%$)	**47 (78.3**$\%$)	19.267	<**0**.**001**
Desire	**50 (83.3**$\%$)	10 (16.7$\%$)	26.667	<**0**.**001**
Personality	**51 (85**$\%$)	9 (15$\%$)	29.400	<**0**.**001**
Consciousness	**49 (81.7**$\%$)	11 (18.3$\%$)	24.067	<**0**.**001**
Pride	22 (36.7$\%$)	38 (63.3$\%$)	4.267	0.039
Embarrassment	34 (56.7$\%$)	26 (43.3$\%$)	1.067	0.302
Joy	**55 (91.7**$\%$)	5 (8.3$\%$)	41.667	<**0**.**001**
*Agency*				
Self-control	26 (43.3$\%$)	34 (56.7$\%$)	1.067	0.302
Morality	34 (56.7$\%$)	26 (43.3$\%$)	1.067	0.302
Memory	36 (60$\%$)	24 (40$\%$)	2.400	0.121
Emotion recognition	**53 (88.3**$\%$)	7 (11.7$\%$)	35.267	<**0**.**001**
Planning	34 (56.7$\%$)	26 (43.3$\%$)	1.067	0.302
Communication	**53 (88.3**$\%$)	7 (11.7$\%$)	35.267	<**0**.**001**
Thought	**45 (75**$\%$)	15 (25$\%$)	15.000	<**0**.**001**
*Subscores*				
Experience subscore	**6**.**95** ± **1**.**88**	4.05 ± 1.88	$-$5.366	<**0**.**001**
Agency subscore	**4**.**68** ± **1**.**91**	2.32 ± 1.91	$-$4.113	<**0**.**001**

Values are expressed in n (frequency) or mean (SD). Significant values after Bonferroni’s correction are reported in bold. Values are shown as frequencies (percentages) or mean ± SD

### Comparison between Authoritarian and Neutral

**Fig. 3:**
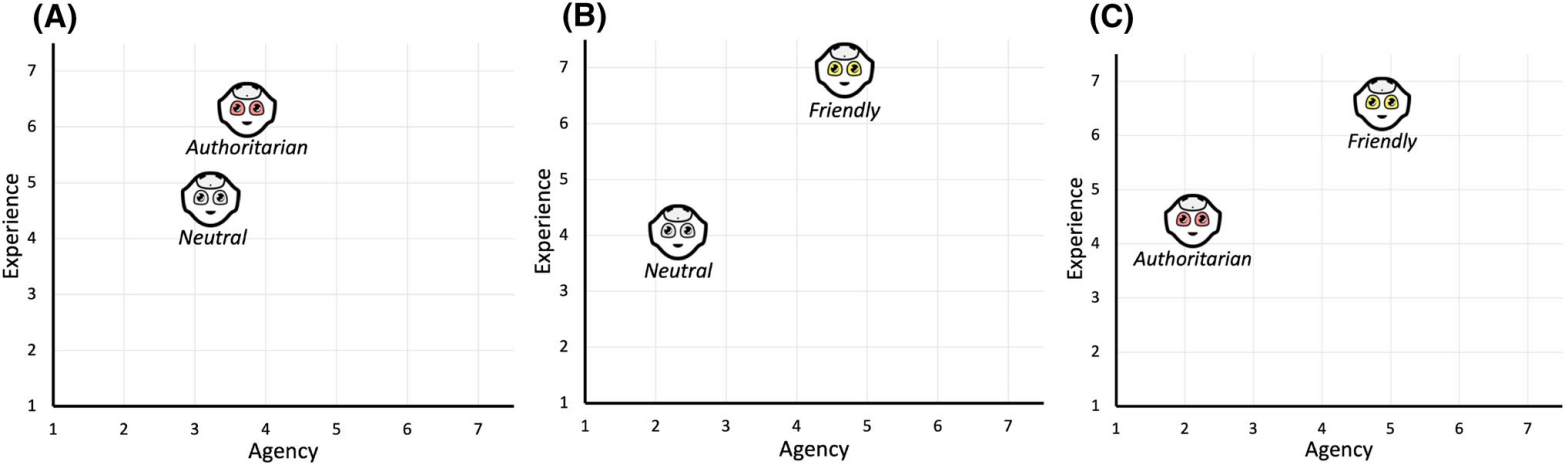
A graphical representation of the experience and agency subscores for the considered pairs. **A** For the *Authoritarian–Neutral* case, we have a statistically significant difference in the Experience subscore (averages 6.30 and 4.70). **B** For the *Friendly-Neutral* case, we have statistically significant differences both on the Experience and the Agency subscores (averages experience 6.95 and 4.05, and averages agency 4.68 and 2.32). **C** For the *Friendly-Authoritarian* case also, we have statistically significant differences both on the Experience and the Agency subscores (averages experience 6.58 and 4.42, and averages agency 4.88 and 2.12)

Forty-three participants (22 males, 21 females; age mean: 43.51, standard deviation: 15.31) completed the survey after observing videos of Neutral and Authoritarian interaction styles. Regarding the Experience dimension of mind, we found that there were statistically significant differences, after applying Bonferroni’s correction, in the judgement of Personality and Consciousness items with more preferences for the Authoritarian style than for the Neutral one (Table 2). No significant difference was found between the observed frequencies on the mental capacities of the Agency dimension (Table 2). Moreover, no significant difference was found in Agency and Experience dimensions between the two interaction styles after we applied Bonferroni’s correction. (Table 2 and Fig. 3A).

### Comparison between Friendly and Neutral

Sixty participants (29 males, 31 females; age mean: 29.00, standard deviation: 6.53) judged the Neutral and Friendly interaction styles on the Dimensions of Mind Perception questionnaire. As for the Experience dimension, we found that more participants considered the Friendly interaction style more capable of experiencing Hunger, Pleasure, Desire, Personality, Consciousness, and Joy than the Neutral one (Table 3). Otherwise, more participants believed that the Neutral interaction style was more capable of experiencing Fear and Rage than the Friendly one (Table 3). Regarding the Agency dimension, more participants believed that the Friendly interaction style was more able in Emotion recognition, Communication, and Thought than the Neutral one (Table 3). Furthermore, the Friendly style obtained significantly higher scores on the Experience and Agency dimensions of ToM compared to the Neutral style (Table 3 and Fig. 3B).

**Table 4:** Comparisons of dimensions of mind perception scores between friendly and authoritarian interaction styles

	Friendly	Authoritarian	$\chi ^2$/Z	*p*
**Experience**				
Hunger	**36 (83.7**$\%$)	7 (16.3$\%$)	19.558	<**0**.**001**
Fear	10 (23.3$\%$)	**33 (76.7**$\%$)	12.302	<**0**.**001**
Pain	9 (20.9$\%$)	**34 (79.1**$\%$)	14.535	<**0**.**001**
Pleasure	**40 (93**$\%$)	3 (7$\%$)	31.837	<**0**.**001**
Rage	4 (9.3$\%$)	**39 (90.7**$\%$)	28.488	<**0**.**001**
Desire	**36 (83.7**$\%$)	7 (16.3$\%$)	19.558	<**0**.**001**
Personality	30 (69.8$\%$)	13 (30.2$\%$)	6.721	0.010
Consciousness	**34 (79.1**$\%$)	9 (20.9$\%$)	14.535	<**0**.**001**
Pride	14 (32.6$\%$)	29 (67.4$\%$)	5.233	0.022
Embarrassment	29 (67.4$\%$)	14 (32.6$\%$)	5.233	0.022
Joy	**41 (95.3**$\%$)	2 (4.7$\%$)	35.372	<**0**.**001**
**Agency**				
Self-control	23 (53.5$\%$)	20 (46.5$\%$)	0.209	0.647
Morality	**32 (74.4**$\%$)	11 (25.6$\%$)	10.256	**0**.**001**
Memory	21 (48.8$\%$)	22 (51.2$\%$)	0.023	0.879
Emotion recognition	**38 (88.4**$\%$)	5 (11.6$\%$)	25.326	<**0**.**001**
Planning	27 (62.8$\%$)	16 (37.2$\%$)	2.814	0.093
Communication	**34 (79.1**$\%$)	9 (20.9$\%$)	14.535	<**0**.**001**
Thought	**35 (81.4**$\%$)	8 (18.6$\%$)	16.953	<**0**.**001**
**Subscores**				
Experience subscore	**6**.**58** ± **2**.**06**	4.42 ± 2.06	$-$ 3.490	<**0**.**001**
Agency subscore	**4**.**88** ± **1**.**71**	2.12 ± 1.71	$-$ 4.187	<**0**.**001**

Values are shown as frequencies (percentages) or mean ± SD. Values are expressed in n (frequency) or mean (SD). Significant values after Bonferroni’s correction are reported in bold

### Comparison Between Friendly and Authoritarian

The survey on the comparison between Friendly and Authoritarian interaction styles was completed by 43 participants (27 males, 16 females; age mean: 26.67, standard deviation: 7.95). We found that more participants considered the Friendly interaction style more capable of experiencing Hunger, Pleasure, Desire, Consciousness, and Joy than the Authoritarian one (Table 4). Conversely, more participants deemed that the Authoritarian interaction style was more capable of experiencing Fear, Pain, and Rage than the Friendly one (Table 4). As for Agency, more participants believed that the Friendly interaction style was more able in Morality, Emotion recognition, Communication, and Thought than the Authoritarian one (Table 4). Moreover, we found that the Friendly style obtained significantly higher scores on the Experience and Agency dimensions of ToM compared to the Authoritarian style (Table 4 and Fig. 3C).

### Dimensions of Mind, Goodspeed-VAD, and Trust

A composite dataset merging the data collected from the three surveys was constructed to analyse the relationship of the Dimensions of Mind subscores with the Goodspeed-VAD variables and Trust. As for the Neutral interaction style, we found that Agency was related to the Perceived Safety subscore of Godspeed-VAD (*n* = 103; $\rho$ = 0.245; *p* = 0.013), whereas no significant association between Experience and Goodspeed-VAD and Trust scores was found. Considering the Authoritarian robot, the Agency was related to the Likeability subscore of Godspeed-VAD (*n* = 86; $\rho$ = 0.213; *p* = 0.049), whereas Experience was not related to Goodspeed-VAD and Trust scores. Finally, Experience and Agency were not associated with Goodspeed-VAD and Trust scores considering the Friendly robot.

## Discussion

Our first hypothesis is confirmed as the results show that the Friendly behaviour was judged more capable of experiencing positive emotions such as Pleasure, Desire, Consciousness, and Joy than the Neutral and Authoritarian; on the contrary, the last one was judged more capable of experiencing negative emotions such as Fear, Pain, Rage, (**H1**) with respect to the Friendly one. We discovered statistically significant differences in Personality with more attribution for both Friendly and Authoritarian interaction styles with respect to the Neutral one. To confirm this, the Neutral was designed to appear as a controlling behaviour and to provide no specific social cues. Interestingly, the Friendly interaction style was judged as more capable to express Hunger than the other two interaction styles. It should be noted that the feeling of hunger is inherently motivating since it recruits positive emotions, namely affective interest, desire, and excitement [29].

Our second hypothesis is also confirmed as the different styles of interaction had a different impact on the perception of the participants on the size of the Agency score (**H2**). In particular, the results showed: the Friendly style scored significantly higher on ToM’s Experience and Agency size than the Neutral style, showing that more participants believed that the Friendly interaction style was more capable of recognising emotions, communicating, and thinking than the Neutral. In addition, the Friendly style scored significantly higher on the Experience and Agency dimensions of ToM compared to the Authoritarian style, showing that more participants believed that the Friendly interaction style was more capable of morality, emotion recognition, communication, and thinking than the Authoritarian one; finally, we have not found any significant difference between Authoritarian and Neutral.

Finally, our third hypothesis is also confirmed as the results show that the perceived mental abilities of the robot are associated with the different characteristics highlighted in the Godspeed questionnaire (**H3**). In particular, it was found: in the Neutral interaction style, the dimension of the agency is related to the perceived security item of the Godspeed questionnaire; in the Authoritarian interaction style the dimension of Agency is related to the Pleasantness item of the Godspeed questionnaire; while in the Friendly interaction style were not found correlations with the items of the Godspeed questionnaire.

On the other hand, no correlations were found between perceived mental abilities and trust. This could be caused by an indirect interaction, as the participants did not interact directly with the robot but observed a human–robot interaction video. In addition, some studies have shown that in human–robot interaction, a cooperative task can increase the level of trust [34]. In [34], the authors demonstrated that the choice of the experimental task could lead to very different results, having a significant impact on the participants’ willingness to follow the robot’s instructions. Consequentially, trust increases the robot’s ability to be accepted as a collaborative partner [18]. Therefore, we should consider that, in our study, the type of cognitive task proposed (Attentive Matrices) by the robot was not cooperative.

## Conclusions

In the HRI field, several studies are starting to investigate whether human beings attribute mental states to a robot while interacting with them. The propensity to recognise non-human agents as capable of having a “mind” can vary depending both on the agent’s external features (e.g., appearance and behaviour) and the observer’s internal dispositions (e.g., beliefs, expectations, motivations, individual differences, and experience). Based on this, the purpose of this study is to evaluate the different impact of three different behavioural styles namely *Friendly, Neutral and Authoritarian*, previously validated [8, 20, 21, 36], on Mind Attribution to the robot with a crowd-sourcing evaluation method enrolling 175 participants aged between 18 and 67 years.

Our results indicate that the personalization of the robot’s interaction style may influence users’ perception of the robot’s mental abilities. More specifically, a Friendly interaction style was judged to be more capable of experiencing positive feelings but also of communicating, thinking, recognising emotions, and behaving morally. These findings further confirm the need to design and validate the robot’s behaviour to facilitate and improve HRI in different application contexts or refrain to use such behavioural interaction styles in the case that mind attribution should be minimised. Indeed, many researchers are starting to evaluate how modelling different behavioural styles of the robot during the interaction may have an impact on performance [21] and acceptance [11]. Hence, our study also contributes to investigating possible reasons behind such differences during the interaction.

The trial was carried out online during the Covid-19 pandemic. For this reason, some of the results obtained, for example, those related to trust were influenced by the protocol adopted. Consequently, we believe that these results can be a starting point for conducting new in-presence experiments. In addition, a future objective is to evaluate the characteristics, of the different interaction styles implemented, which can influence the level of trust, using a cooperative task and through direct human–robot interaction.
